# Supplementary material for: Bioinspired thermadapt shape-memory polymer with light-induced reversible fluorescence for rewritable 2D/3D-encoding information carriers
Source: Nat Commun. 2023 Nov 6;14:7131. doi: 10.1038/s41467-023-42795-1 (PMC10628284; doi:10.1038/s41467-023-42795-1)
Supplement: Supplementary file 1 — Supplementary Information [file 41467_2023_42795_MOESM1_ESM.pdf]

Supplementary Information

Bioinspired thermadapt shape-memory polymer with light-induced reversible fluorescence for rewritable 2D/3D-encoding information carriers

Jinhui Huang[1, 2], Yue Jiang[1, 2], Qiuyu Chen[1, 2], Hui Xie[1, 2, *], Shaobing Zhou[1, 2, *]

[1]Institute of Biomedical Engineering, College of Medicine, Southwest Jiaotong University, Chengdu 610031, China.

[2]Key Laboratory of Advanced Technologies of Materials Ministry of Education, School of Materials Science and Engineering, Southwest Jiaotong University, Chengdu 610031, China.

*Corresponding authors.

Email: huixie@swjtu.edu.cn (H.X.); shaobingzhou@swjtu.edu.cn (S.Z.)

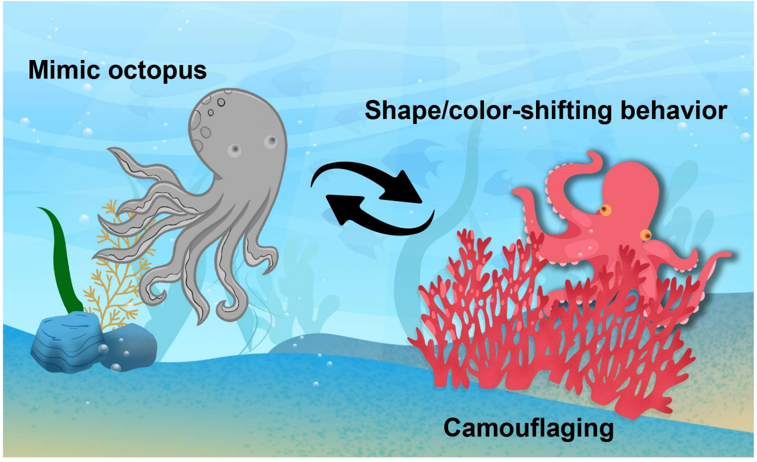

Supplementary Fig. 1 Illustration of the biomimicry of the mimic octopus. The photo describes a mimic octopus camouflage as a coral by shape/color-shifting.

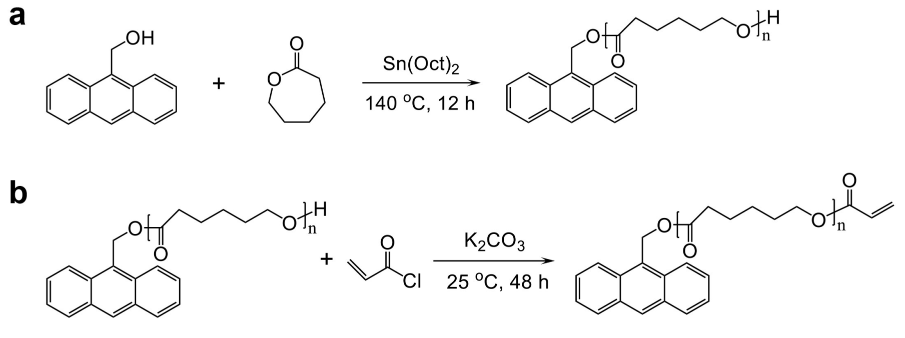

Supplementary Fig. 2 Synthesis route of photoactive material AC-PCL-AN. a The synthesis of PCL-AN via the ring-opening polymerization of the ε-Caprolactone by 9-Anthracenemethanol, **b** The synthesis of AC-PCL-AN by modifying PCL-AN by acryloyl chloride.

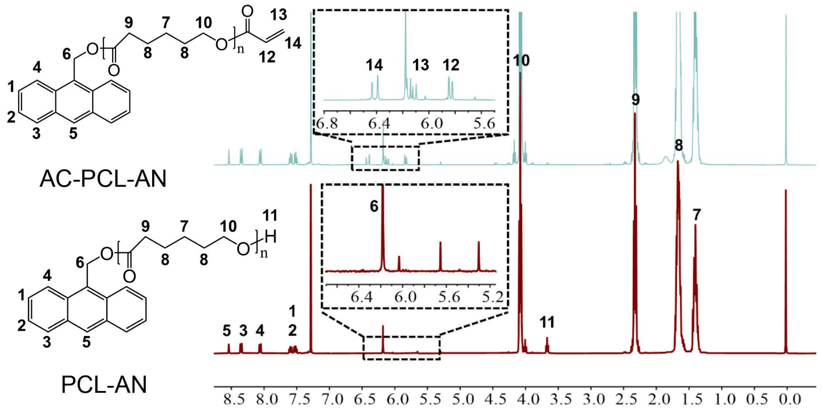

Supplementary Fig. 3 ^{1}H NMR characterization of PCL-AN and AC-PCL-AN. The ^{1}H NMR spectra were recorded by AVANCE NEO 400 MHz spectrometer (Bruker, Germany) at room temperature using $CDCl_3$ as the solvent and tetramethylsilane as the internal reference. The characteristic peaks of PCL units appear at 1.39 ppm, 1.65 ppm, 2.31 ppm, and 4.06 ppm, and the characteristic peaks of the anthracene ring appear in the range of 7.5-8.6 ppm. For AC-PCL-AN, additional characteristic peaks at 5.85 ppm, 6.10 ppm, and 6.44 ppm demonstrate the existence of CH_2=CH-.

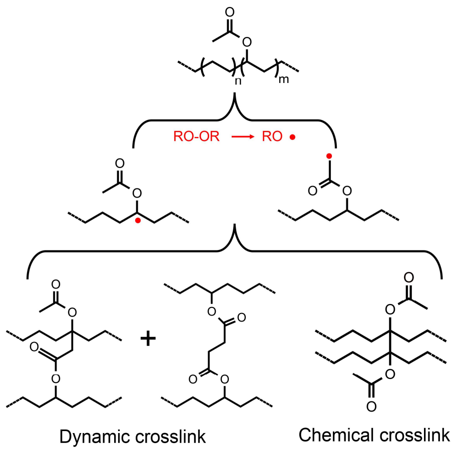

Supplementary Fig. 4 Mechanism of the cross-linking reaction for preparing cEVA. EVA produces three types of cross-linking in the presence of the initiator DCP, one of which is a chemical cross-linking because of the formation of C-C bonds. The other two types contain ester groups and may undergo potential ester exchange reactions.[1]

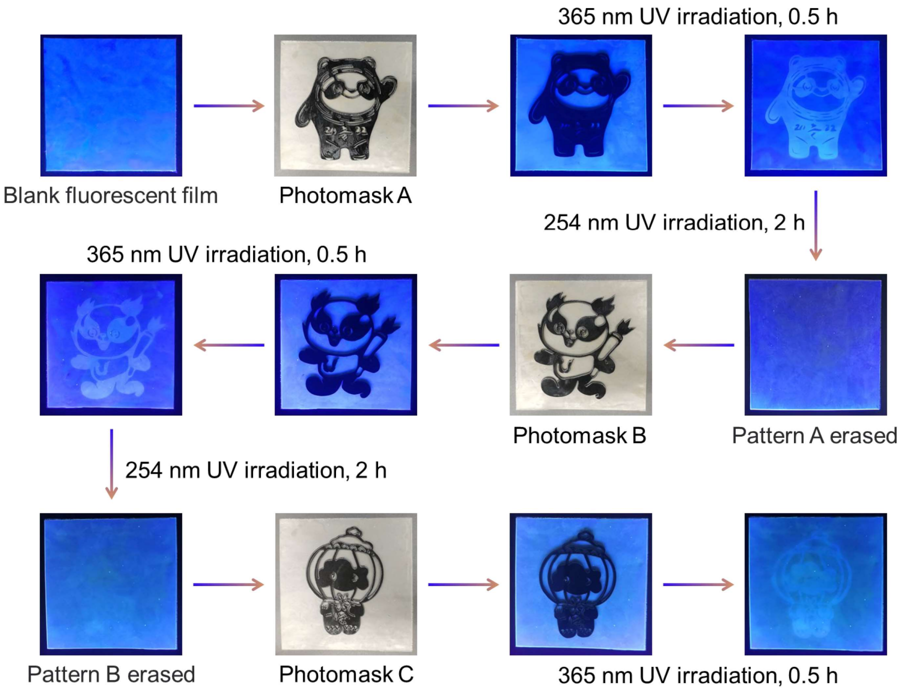

Supplementary Fig. 5 Photos showing creation/erasure cycles of the 2D fluorescent patterns by alternate 365 nm and 254 nm UV irradiation. Pattern A: Bing Dwen Dwen, Pattern B: Rongbao, Pattern C: Shuey Rhon Rhon.

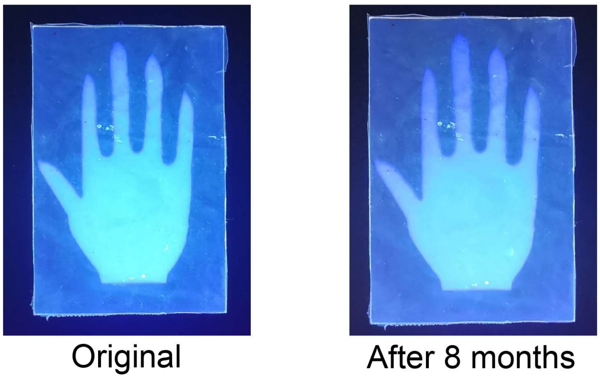

Supplementary Fig. 6 Demonstration of long-term stability of the patterned TSFF. The sample was stored in an ambient environment for 8 months.

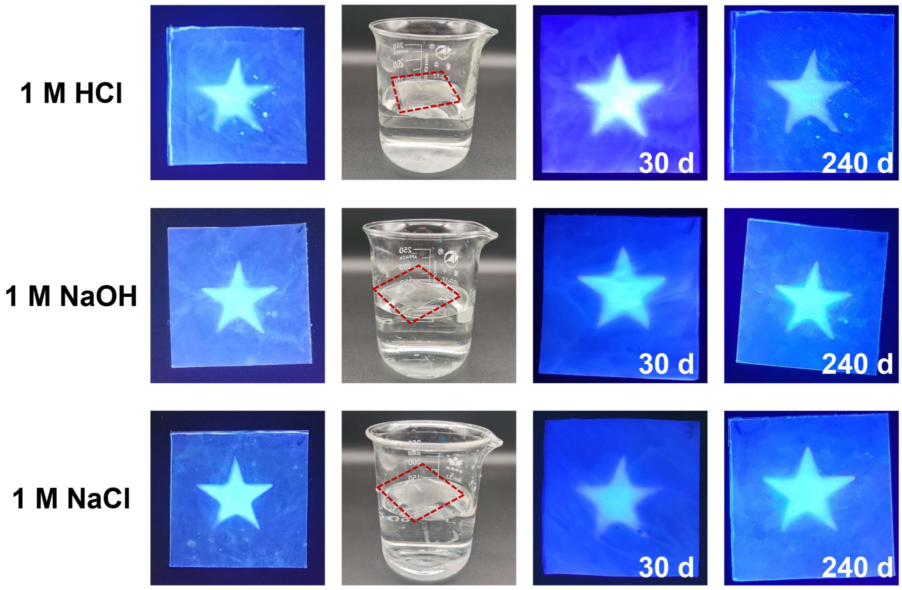

Supplementary Fig. 7 Demonstration of chemical stability of the patterned TSFF. The sample was immersed in strong acidic (HCl), strong base (NaOH), and salt solution (NaCl) at ambient temperature for 240 days, respectively.

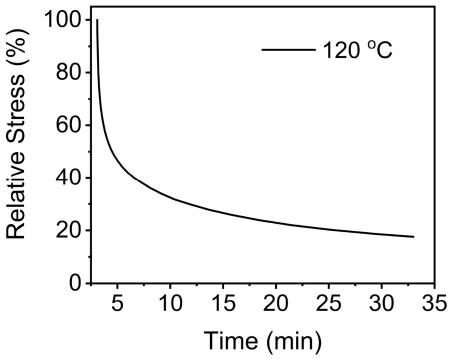

Supplementary Fig. 8 Stress relaxation curve of the pristine cEVA sample. The sample contains TBD catalyst but without adding AC-PCL-AN.

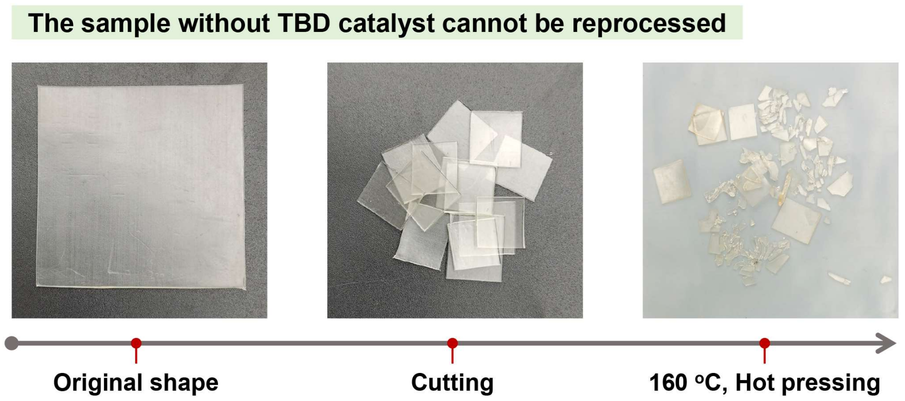

Supplementary Fig. 9 Investigation of the reprocessing property of the sample without TBD. The controlled sample without TBD catalyst cannot be reprocessed.

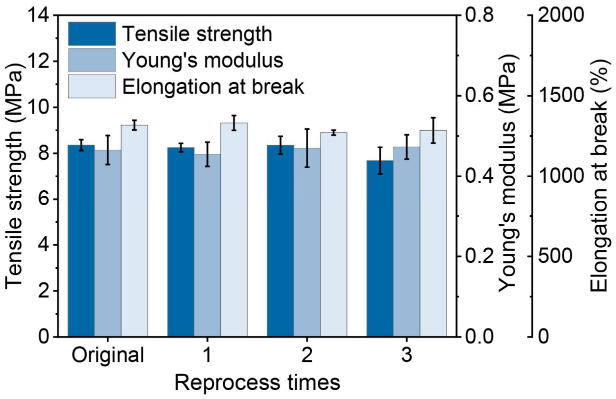

Supplementary Fig. 10 Comparison of the mechanical properties between the original and reprocessed TSFF samples. The results show that Young's modulus, tensile strength, and elongation at break of the reprocessed TSFF sample are almost identical to those of the original sample with only a very small variation. Error bars represent the standard deviation calculated by the data sets ($n = 3$).

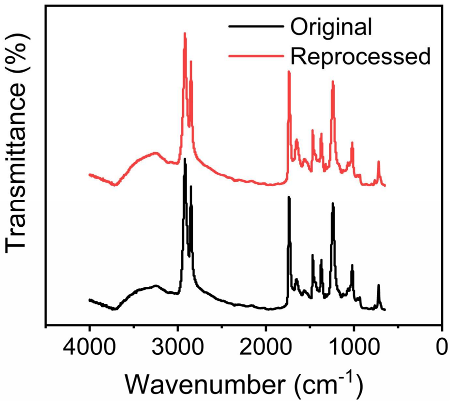

Supplementary Fig. 11 FT-IR spectra of the original and reprocessed TSFF samples. The chemical structure of the reprocessed sample show no significant changes compared with the original sample.

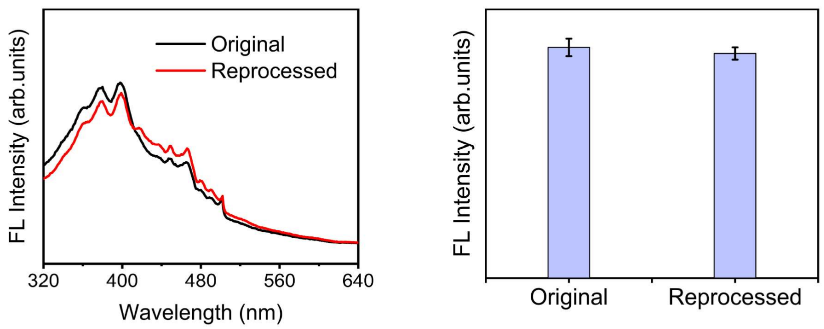

Supplementary Fig. 12 Fluorescence intensity of the original and reprocessed TSFF samples. The fluorescence intensity of the reprocessed sample shows no significant change compared with the original sample. Error bars represent the standard deviation calculated by the data sets (n = 3).

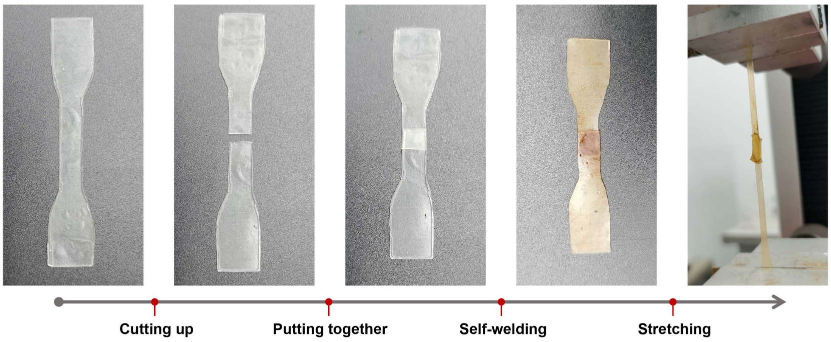

Supplementary Fig. 13 The self-welding process of TSFF. A dumbbell-shaped TSFF specimen was cut into two pieces, followed by partly overlaying and hot-pressing for welding. The welded sample is highly stretchable.

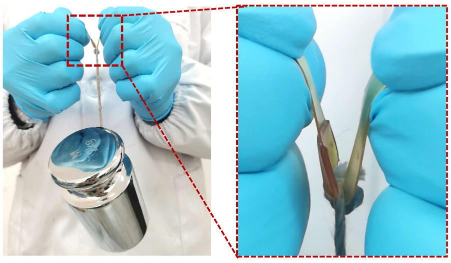

Supplementary Fig. 14 Demonstration of the excellent welding properties of TSFF. The Photograph shows that a self-welded sample that can hold a 2 kg weight.

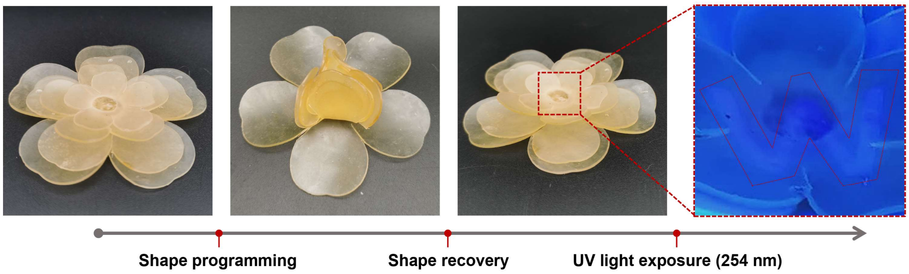

Supplementary Fig. 15 Photograph of the 3D flower produced by welding several petal-like TSFF samples and its shape-memory properties. The 2D information "W" was printed in the center.

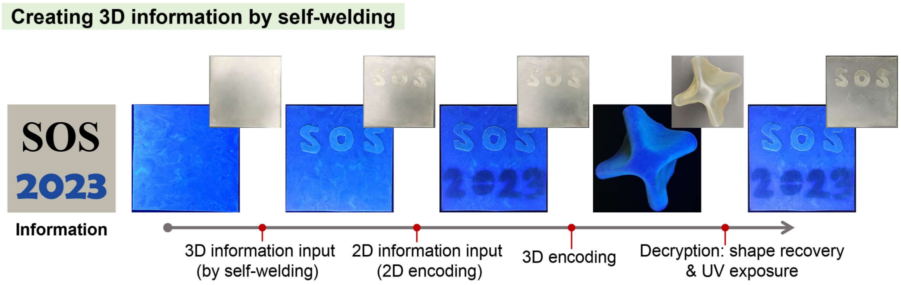

Supplementary Fig. 16 The proof-of-concept of creating 3D information by self-welding.

The 3D information "SOS" is created by welding three pieces of TSFF with the substrate.

Supplementary Table 1. Comparison of TSFF with recently reported anti-counterfeit materials.

Material	Encoding		Information encryption/decryption		Substrate	Mechanism	Self-welding	Reprocessability	Ref.
	2D	3D	Stimulus	Repeatability					
P(DMA-DEAN)/P(SMA-9-ANA)	Yes	No	Temperature, UV light	Yes	Hydrogel	Shape-memory and fluorescence	No	No	2
AOP thermoset	No	Yes	Temperature, water	Yes	Thermoset	Shape-memory and hydrophobic interactions	No	Yes	3
3D printing hydrogel	Yes	No	UV light	No	Hydrogel	Fluorescence	No	No	4
Photonic crystal films	Yes	No	White light, UV light, and darkness	No	Film	Structural, fluorescent, and phosphorescent color	No	No	5
P(eDEGA-coOEGMA)/ZnS@SiO2	Yes	No	Temperature	No	Film	Thermochromic	No	No	6
PAA-*g*-PLLA/TPE-PDLA	Yes	No	UV light	Yes	Hydrogel	AIE fluorescence	No	No	7
Donor-acceptor Stenhouse adducts	Yes	No	Temperature, UV light, white light, water	No	Gel	Photochromic, thermochromic, hydrochromic	No	No	8
Azo-polymers	Yes	No	Temperature, UV light	Yes	Film	Photonic structures and chromatic polarization	No	Yes	9
P(VI-co-MAAc)/VPTP	Yes	No	Temperature, UV light	Yes	Hydrogel	Shape-memory and fluorescence	No	No	10
PVA-C6/P(AAm-co-AAc)	No	Yes	Temperature	Yes	Hydrogel	Shape-memory	No	No	11
Fluorescent dyes inks	Yes	No	pH, metal ions, and polymer rigidity	Yes	Paper	Fluorescence	No	No	12
cEVA/AC-PCL-AN	Yes	Yes	Temperature, UV light	Yes	Polymer film	Thermadapt shape-memory and fluorescence	Yes	Yes	**This work**

Supplementary References

1. Gao, Y., Liu, W. & Zhu, S. Reversible shape memory polymer from semicrystalline poly (ethylene-co-vinyl acetate) with dynamic covalent polymer networks. *Macromolecules* **51**, 8956-8963 (2018).
2. Shang, H., et al. Integrating photorewritable fluorescent information in shape-memory organohydrogel toward dual encryption. *Adv. Opt. Mater.* **10**, 2200608 (2022).
3. Yang, X., et al. A repeatable dual-encryption platform from recyclable thermosets with self-healing ability and shape memory effect. *Adv. Funct. Mater.* **32**, 2205177 (2022).
4. Chen, D., et al. Orthogonal photo-chemistry towards direct encryption of a 3D printed hydrogel. *Adv. Mater.* **35**, 2209956 (2023).
5. Huang, H., Li, H., Yin, J., Gu, K., Guo, J. & Wang C. Butterfly-inspired tri-State photonic crystal composite film for multilevel information encryption and anti-counterfeiting. *Adv. Mater.* **35**, 2211117 (2023).
6. Wu, Y., Sun, R., Ren, J., Zhang, S. & Wu, S. Bioinspired dynamic camouflage in programmable thermochromic-patterned photonic films for sophisticated anti-counterfeiting. *Adv. Funct. Mater.* **33**, 2210047 (2023).
7. Lan, X., et al. Multi-level information encryption/decryption of fluorescent hydrogels based on spatially programmed crystal phases. *Small* **19**, 2205960 (2023).
8. Dong, Y., et al. Harnessing molecular isomerization in polymer gels for sequential logic encryption and anticounterfeiting. *Sci. Adv.* **8**, eadd1980 (2022).
9. Xu, W.C., Liu, C., Liang, S., Zhang, D., Liu, Y. & Wu, S. Designing rewritable dual-mode patterns using a stretchable photoresponsive polymer via orthogonal photopatterning. *Adv. Mater.* **34**, 2202150 (2022).
10. Zhu, C.N., et al. Dual-encryption in a shape-memory hydrogel with tunable fluorescence and reconfigurable architecture. *Adv. Mater.* **33**, 2102023 (2021).
11. Lu, H., et al. Programming shape memory hydrogel to a pre-encoded static deformation toward hierarchical morphological information encryption. *Adv. Funct. Mater.* **32**, 2206912 (2022).
12. Liu, J., et al. C_3-symmetric propeller-like phenanthridine derivative with multiple write-in modes for programmable anti-counterfeiting. *Chem. Mater.* **34**, 9492-9502 (2022).
