# Supplementary material for: Bioinspired thermadapt shape-memory polymer with light-induced reversible fluorescence for rewritable 2D/3D-encoding information carriers
Source: Nat Commun. 2023 Nov 6;14:7131. doi: 10.1038/s41467-023-42795-1 (PMC10628284; doi:10.1038/s41467-023-42795-1)
Supplement: Supplementary file 3 — Description of Additional Supplementary Files [file 41467_2023_42795_MOESM3_ESM.pdf]

Description of Additional Supplementary Files

File Name: Supplementary Movie 1

Demonstration of thermally induced plasticity of TSFF

File Name: Supplementary Movie 2

Shape recovery behavior of TSFF in successive shape-memory cycles
